# Three-Dimensional Printing of Yttrium Oxide Transparent Ceramics via Direct Ink Writing

**DOI:** 10.3390/ma17133366

**Published:** 2024-07-08

**Authors:** Qiming Chen, Huibing Li, Weijie Han, Jian Yang, Wentao Xu, Youfu Zhou

**Affiliations:** 1 Key Laboratory of Optoelectronic Materials Chemistry and Physics, Fujian Institute of Research on the Structure of Matter, Chinese Academy of Sciences, Fuzhou 350002, China; 2 Fujian Science & Technology Innovation Laboratory for Optoelectronic Information of China, Fuzhou 350108, China; 3 University of Chinese Academy of Sciences, Beijing 100083, China

**Keywords:** transparent ceramic, 3D printing, yttrium oxide, DIW

## Abstract

The utilization of 3D printing technology for the fabrication of intricate transparent ceramics overcomes the limitations associated with conventional molding processes, thereby presenting a highly promising solution. In this study, we employed direct ink writing (DIW) to prepare yttrium oxide transparent ceramics using a ceramic slurry with excellent moldability, solid content of 45 vol%, and shear-thinning behavior. A successfully printed transparent yttrium oxide ring measuring 30 mm in diameter, 10 mm in inner diameter, and 0.9 mm in thickness was obtained from the aforementioned slurry. After de-binding and sintering procedures, the printed ceramic exhibited in-line transmittance of 71% at 850 nm. This work not only produced complex yttria transparent ceramics with intricate shapes, but also achieved in-line transmittance that was comparable to that of the CIP method (79%), which can meet certain optical applications.

## 1. Introduction

Known also as 3D printing, additive manufacturing (AM) is a type of manufacturing technique that was invented in the 1980s that creates solid objects with intricate geometric features by layering small layers of powder, liquid, or solid. In contrast to conventional reduction manufacturing, which involves drilling and milling, additive manufacturing eliminates the need for molds [1]. The model should be designed in CAD and then imported into the 3D printer. The process of deposition of materials using printing heads, nozzles, lasers, and other forming technologies results in less waste of raw materials, a shorter and simpler manufacturing process, and the ability to form complex shapes and gradient structures [2,3,4]. A novel technology that combines computer modeling, mechanical control, and material science is additive manufacturing. Fused deposition modeling technology (FDM) [5], powder bed fusion technology (PBF) [6], laser direct metal deposition forming technology (DMD) [7], the stereolithography-forming process (SLA) [8,9], and other technologies are examples of additive manufacturing [10,11,12,13,14].

Direct ink writing (DIW) is a low-cost, quick-forming additive manufacturing process available today for 3D printing [15]. In DIW processes, the printing slurry is extruded via a needle, and the printed components are generated by stacking the printing layers in line with the computer-set model, controlled by the G-code file. The complex ceramic examples that are not manufactured by conventional CIP are obtained after further processing, such as calcination, sintering, and annealing.

It has been widely reported that DIW can be used to 3D print transparent ceramics, including AlON [16], YAG [17], Al_2_O_3_ [18], and others [19,20]. Nevertheless, 3D printing yttria transparent ceramics has received less attention. As a transparent ceramic, yttria has found wide use. Yttria transparent ceramics find extensive applications in the field of optics, serving as transparent window materials and finding utility in laser devices, optical sensors, and artificial joints. The main techniques used in the production of yttria transparent ceramic consist of vacuum sintering, gel casting [21], hot isostatic pressing (HIP) [22], and spark plasma sintering (SPS) [23]. Alternative methods include dry pressing and cold isostatic pressing (CIP). While all of these methods can offer high-grade yttria transparent ceramics, they are either not able to create complicated constructs or do so at the cost of additional tools and processes. Owing to advances in 3D printing, it is now possible to manufacture high-transparency yttria ceramic materials that can be quickly and efficiently shaped into novel shapes.

In this study, we develop a self-coagulating ceramic slurry with a solid content of 45 vol% and shear-thinning rheological properties, which is suitable for direct ink writing (DIW) to fabricate ceramic green bodies with intricate shapes. Subsequently, the 3D-printed ceramic green bodies with complex shapes underwent drying, de-binding, and vacuum sintering processes to achieve in-line transmittance of 71% at 850 nm. The densification mechanism was also investigated to understand the evolution of density and microstructure during the sintering process.

## 2. Materials and Methods

### 2.1. Materials

The Y_2_O_3_ ceramic powder (JiaHua, Jiangyin, China, 5.2 μm, 2.4 m^2^/g, 99.99%) was selected, as were ZrO_2_ (Adamas-beta, Shanghai, China, 2.6 μm, 3.9 m^2^/g, 99.9%) and La_2_O_3_ (Macklin, Shanghai, China, 50 nm, 99.99%). The powder mixture was weighed according to the ratio of Y_1.74_Zr_0.2_La_0.06_O_3_, mixed with ethanol, and ground with a high-purity zirconia mill ball (diameter: 2 mm) for 24 h. Subsequently, it was dried and passed through a 200-mesh sieve. The average particle size (D_50_) of the powder was measured as 1.2 μm, with a specific surface area (SSA) of 7.9 m^2^/g. The additives used in this work included dispersant Isobam-104 as well as auxiliary dispersant triammonium citrate TAC. Glycerin served as both a water-retaining agent and a lubricant, while deionized water was employed as the solvent for dissolving various additives and ceramic powders.

### 2.2. Slurry Preparation

This study aims to formulate a ceramic slurry suitable for 3D printing, which is characterized by low organic content, high solid content, and excellent formability. The rheological properties and zeta potential of the dispersant at low solid content were investigated to determine its optimal dosage. For instance, appropriate utilization of Isobam-104 ensures optimal dispersion and facilitates the preparation of a high-solid-content slurry [24,25,26]. Furthermore, the incorporation of TAC can effectively delay the spontaneous gelation process of Isobam-104 and enhance the dispersion performance of the slurry to a certain extent [27]. Glycerol, possessing three hydroxyl groups that form hydrogen bonds with water molecules, exhibits retardation in the water evaporation rate, thereby maintaining slurry moisture.

A slurry with a solid content of 15.7 vol%, consisting of Isobam-104 at a concentration range of 0.3–0.9 wt%, was prepared. Its relative viscosity was evaluated to determine the dispersant concentration, while the influence of pH on its potential was investigated to identify the optimal pH for the slurry. Additionally, the rheological characteristics of three different slurries with solid contents of 40 vol%, 45 vol%, and 47 vol% were analyzed to optimize their parameters.

**Figure 1:**
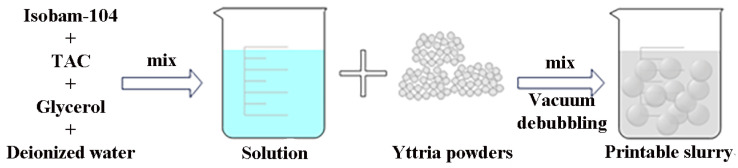
The process of preparing printable slurry.

Once the optimal ratio of additive (Isobam-104: 0.7 wt%; TAC: 0.5 wt%) to solid content (45 vol%) was determined, the deionized water and the additive were transferred into a beaker. The mixture was stirred using a magnetic stirrer for 12 h, after which the magneton was removed. After adding the powder, continuous stirring was maintained for 30 min. The mixture was subsequently transferred into a vacuum defoaming machine and defoamed at speeds of 500 rpm and 2000 rpm for durations of 1.5 min and 20 s, respectively. Immediately, the resulting paste was poured into a material bottle for printing. Figure 1 illustrates the process of preparing printable slurry.

### 2.3. 3D Printing of Slurry

**Figure 2:**
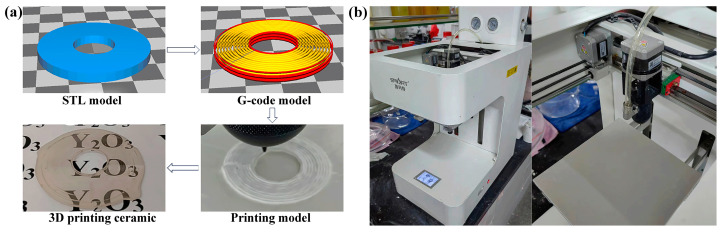
(**a**) The construction of the print model; (**b**) 3D printer.

The 3D model was designed using AutoCAD (2019) software and exported in the STL file format, which was subsequently converted into G-code format through the Smart 3D slicing (1.4) software. The resulting model was then imported into the 3D printer for fabrication based on the design specifications. Figure 2a illustrates both the STL model generated by AutoCAD and the corresponding G-code representation obtained after slicing. Utilizing a Smart 3D printer, a ceramic green body was printed by moving the printing needle along fixed *x* and *y* axes within a plane of constant height, while simultaneously adjusting the *z*-axis position through controlled movements of the print platform. The printing needle had a diameter of 1.0 mm, while the filling density (also known as line spacing) was set at 100% and the line extrusion rate (printing speed) remained constant at 7 mm/s, as illustrated in Figure 2b of the 3D printer. After pouring the slurry into the material bottle, it was connected to the printing needle at the front and to an air compressor at the back. The slurry was then extruded through the printing needle by pushing the piston using air pressure. Following printing, the green body is detached from the printing platform and left to dry under ambient temperature and atmospheric pressure for 24 h [18].

### 2.4. De-Binding and Sintering Processing

In the process of de-binding, the combustion of organic additives enables the acquisition of a pure ceramic green body. To establish an efficient de-binding system, TG analysis was conducted on the green bodies. After the drying process, the ceramic green body was subjected to a temperature of 700 °C in a Muffle furnace for a duration of 3 h to eliminate organic components. The sintering process was carried out using a vacuum sintering furnace at temperatures of 1750 °C, 1800 °C, and 1850 °C for a period of 8 h each. Subsequently, the ceramic underwent annealing in a Muffle furnace at a temperature of 1250 °C for 6 h. Through de-binding, sintering, and annealing processes, an allotrope with high density and transparency was successfully achieved.

### 2.5. CIP Method

Cold isostatic pressing (KJYc300, Jinkaiyuan, Taiyuan, Shanxi, China) was used as a traditional preparation method for ceramics in comparison to 3D printing. After the powder was pressed into round discs, the resulting discs ere held in the CIP chamber at 200 MPa for 2 min. Further processing was consistent with the sample processing for 3D printing.

### 2.6. Materials Characterization

The zeta potential was measured using a BI-200SM instrument (Brookhaven, NJ, USA), while the pH was adjusted utilizing an NH_3_·H_2_O solution. The impact of the dispersant on the slurry viscosity was evaluated by employing an NDJ-8S viscometer (Pingxuan, Shanghai, China) equipped with a No. 3 rotor. Furthermore, the rheological properties of the ceramic slurry were investigated using a Dynamic Hybrid Rheology 2 (TA Instrument, Shanghai, China) rotational rheometer with a parallel plate diameter of 25 mm.

The TG analysis of the sample was conducted using a NETZSCH STA449K instrument (Netzsch, Bavaria, Germany) with a temperature range of 20–1000 °C and a heating rate of 10 °C/min. The sectional microstructure of ceramic samples was observed via scanning electron microscopy (Gemini 360, Zeiss, Oberkochen, Germany). Phase analysis of ceramics was performed using an X-ray diffractometer (XRD, Miniflex-600, Rigaku, Japan) with CuKα1 radiation in the 2θ range of 10–80°. In-line transmittance measurements were carried out on polished samples with thicknesses of 0.9 mm using a Lambda950 Fourier-transform infrared (FT-IR, Perkin Elmer, Shanghai, China) spectrometer within the wavelength range of 200–850 nm.

## 3. Results and Discussion

### 3.1. Slurry Design

The high solid content and optimal rheological properties of the slurry play a pivotal role in the ceramic preparation process in DIW. The appropriate content of dispersant can effectively disperse the powder in the slurry, thereby reducing the viscosity and promoting the preparation of high-solid-content slurry with improved uniformity. Moreover, the solid content factor significantly influences both the viscosity and the modulus of the slurry, consequently impacting print molding effects. This chapter will discuss the selection of dispersant content and solid content.

#### 3.1.1. The Effect of Dispersants on Powder Suspension

If the powder in the slurry is not effectively dispersed, it can greatly impact the material’s molding effect and lead to poor stability of the powder within the slurry [28]. This can result in agglomeration and settling of the powder, leading to extremely unstable rheological properties of the slurry [29]. The incorporation of additives facilitates the adsorption of the polymer onto the surface of powder particles, enhancing their surface charge potential and promoting mutual repulsion among particles. Simultaneously, the presence of polymer chains attached to the particle surfaces induces a steric hindrance effect, impeding particle reaggregation. The dispersant Isobam-104 is highly suitable for enhancing particle stability in slurry, facilitating the preparation of high-solid-content slurries and thereby improving ceramic density. Additionally, it exhibits a spontaneous coagulation effect during late-stage preparation, leading to the formation of a gel network between particles and consequently enhancing the energy storage modulus of the slurry. Hence, it is well-suited for DIW applications. In order to facilitate slurry preparation, triammonium citrate is incorporated as an auxiliary dispersant in this study. This dispersant not only enhances particle dispersion within the slurry, but also improves the gel formation barrier of Isobam-104, thereby enabling the production of high-solid-content slurries [27]. 

**Figure 3:**
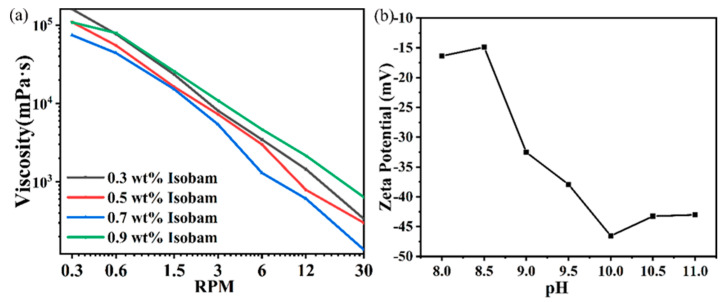
(**a**) Viscosity curve for the slurry with different Isobam-104 content; (**b**) zeta potentials at different pH values.

In the 15.7 vol % slurry, the Isobam-104 content ranges from 0.3 to 0.9 wt%. The viscosity analysis is presented in Figure 3a. According to the viscosimeter analysis, an increase in Isobam-104 content leads to enhanced polymer chain attachment on particle surfaces, resulting in steric hindrance effects that promote the dispersion of particles within the slurry. The viscosity of the slurry is generally at its minimum when the addition amount of Isobam-104 is 0.7 wt%. However, as the additional amount of Isobam-104 reaches 0.9 wt%, the viscosity of the slurry increases due to saturation of polymer chain adsorption on particle surfaces. The introduction of additional additive polymers leads to intertwinement within the slurry, resulting in a significant rise in viscosity [26]. Therefore, it is recommended to incorporate Isobam-104 at a concentration of 0.7 wt% as an additional component. However, the addition of Isobam-104 alone does not suffice for slurry stabilization [30]. Previous studies have encountered challenges in achieving high solid content during the preparation process of Isobam-104 slurry, with only 0.7 wt% being attainable thus far. This difficulty primarily stems from the rapid spontaneous coagulation of Isobam-104 in an air environment upon powder addition, leading to a sudden surge in slurry viscosity and impeding the preparation process. Lu et al. demonstrated that the incorporation of TAC can effectively retard the rate of spontaneous coagulation formation for Isobam-104 [27], and Zhang L et al. also proposed that the addition of ammonium citrate can significantly enhance the slurry’s dispersibility [30], with a TAC content utilized in this study at 0.5 wt%. The slurry pH is adjusted to 8–10 using NH_3_·H_2_O due to the easy hydrolysis of Y_2_O_3_ in acidic and weakly alkaline environments, as depicted by its zeta potential shown in Figure 3b. When the system pH exceeds 8, the particle surface exhibits a negative ζ potential; at a pH of 10.0, this potential measures −46.5 mV. Consequently, electrostatic repulsion prevents particle agglomeration, ensuring optimal slurry stability at pH 10.0.

#### 3.1.2. Effect of Different Solid Loading on Slurry Rheology

The solid content in DIW plays a crucial role in influencing the rheological properties of slurry and the density of ceramics after sintering, with a direct impact on the slurry viscosity. For successful DIW, it is essential for the slurry to exhibit shear-thinning behavior, enabling low-viscosity extrusion at high shear rates during printing while maintaining high viscosity post-extrusion to prevent spreading and ensure proper layer stacking for optimal green body formation [25,31]. To achieve this, Isobam-104, TAC, and glycerin were added to deionized water as solvents, while ceramic powder was incorporated at volumetric ratios of 40%, 45%, and 47% to obtain highly viscous ceramic slurries.

**Figure 4:**
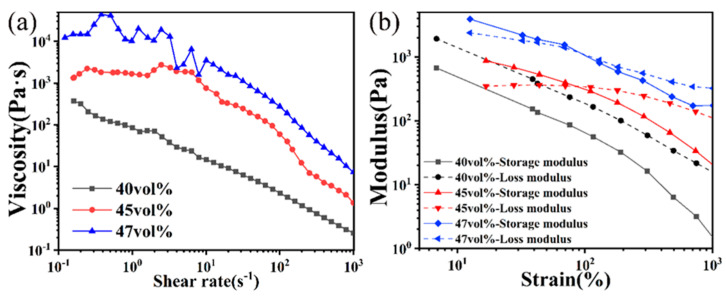
Rheological properties of the print slurry with varying solid loading: (**a**) viscosity evolution and (**b**) complex shear strain.

The influence of the powder solid content on the viscosity and printability of ceramic slurry was investigated using a shear rheological curve. The rheological curve depicts the relationship between viscosity and shear rate (Figure 4a). Generally, slurry with varying solid contents exhibited shear-thinning behavior. It is evident that an increase in solid loading leads to an increase in slurry viscosity. Notably, when the solid loading increased from 45 vol% to 47 vol%, the viscosity at low shear rates (10^−1^–100 s^−1^) for 47 vol% was approximately ten times higher than that of 45 vol%. If the viscosity was excessively high, it impeded the smooth extrusion of the slurry. Conversely, a solid content of 40 vol% and 45 vol% resulted in insufficient and moderate viscosities, respectively. To gain further insights into the molding effect of the slurry, Figure 4b illustrates modulus analysis for three different solid-content slurries subjected to strains ranging from 10 to 1000%. The loss modulus of the 40 vol% slurry consistently surpassed its storage modulus within this strain range, indicating that the slurry remained fluid and could be extruded during printing. However, it tended to collapse and spread after extrusion, which hindered the proper formation of ceramic green bodies. Under low strain, the storage modulus of a 45 vol% slurry surpassed the loss modulus, indicating its solid paste-like nature within this range, which corresponded to the state of stacked slurry layers after extrusion. However, when the strain exceeded 90%, the loss modulus became greater than the storage modulus, suggesting fluid behavior in this range (>90%). Consequently, it can be inferred that a 45 vol% slurry is suitable for direct ink writing (DIW) applications. The slurry with a solid content of 47 vol% exhibited similar rheological properties to that with a 45 vol% content. It demonstrated solid behavior under low strain conditions and transitioned into a fluid state at high strain rates. However, its viscosity was excessively high, which hindered the preparation process and compromised the uniformity of the slurry. During the subsequent printing process, upon extrusion from the print needle, the print slurry underwent spontaneous coagulation within 5–10 s, resulting in a surge in viscosity and enabling the printed lines to maintain their shape without collapsing. In conclusion, a 45 vol% slurry proved to be the most suitable for direct ink writing (DIW) applications; thus, it was utilized and printed in this study.

### 3.2. De-Binding and Sintering Process

The slurry formulation for printing transparent ceramic green bodies consisted of 45 vol% powders and 0.7 wt% Isobam-104, as determined by experimental investigations.

#### 3.2.1. Determination of De-Binding Procedure by TGA

**Figure 5:**
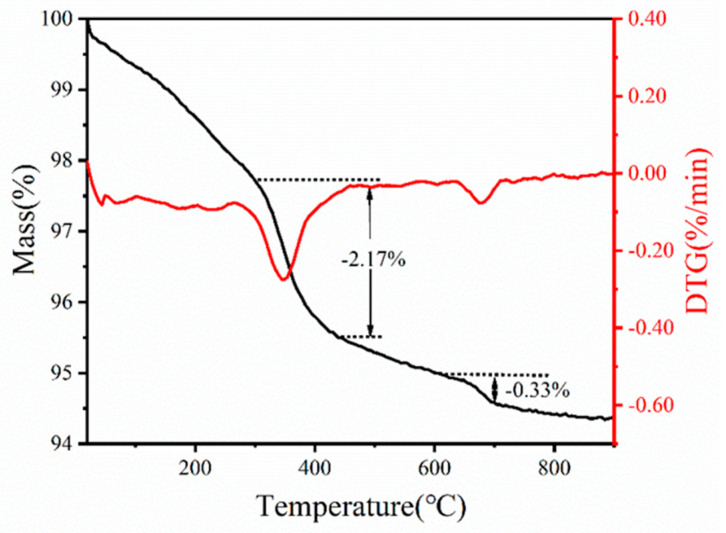
TG analysis curves of 3D-printed green body from slurry with 45 vol% solid loading and 0.7 wt% Isobam-104, 0.5 wt% TAC, 1.2 wt% glycerol.

**Figure 6:**
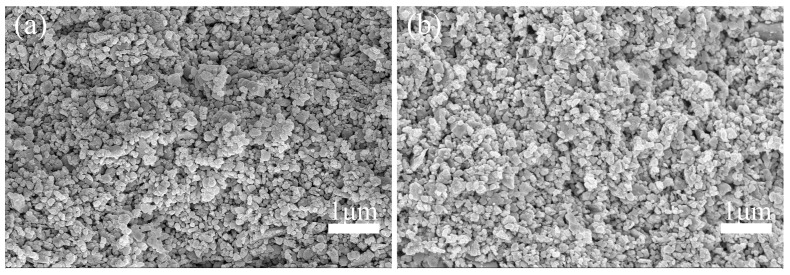
Microstructure of the 3D-printed green body was examined (**a**) prior to de-binding and (**b**) after de-binding at a temperature of 700 °C for a duration of 3 h.

To facilitate temperature selection during de-binding, the changes in mass and composition of the printed green body were analyzed using Thermogravimetry. The TGA curve depicted in Figure 5 exhibits a two-step thermal gradient: (1) water loss (20–250 °C) and (2) removal of organic additives (250–450 °C). In the first step, the TG curve reveals that, within the low temperature range (20–250 °C), mass loss primarily occurs due to water evaporation, TAC decomposition, and NH_3_ volatilization. Subsequently, in the second step, both TG and DTG curves demonstrate significant mass loss associated with the decomposition of glycerol and Isobam-104 decompositions. The decomposition process of organic matter concludes at 450 °C, followed by a slight mass loss attributed to the volatilization of bound water within the green body. The marginal mass reduction observed at 650 °C can be ascribed to the rapid heating rate (10 °C/min) employed in the thermal analysis experiment, which prevents the complete expulsion of carbon and water decomposed by organic additives from the interior [32]. Considering the aforementioned factors, it is advisable to adopt a slower heating rate for this system in order to ensure the complete burnout of the polymer. Consequently, the green body was subjected to calcination at 700 °C for 3 h in an air atmosphere with a heating rate of 2 °C/min, aiming to guarantee thorough combustion. As depicted in Figure 6a, the interior of the green body exhibited remarkable uniformity with minimal agglomeration and only a few residual pores after calcination, thereby confirming the successful elimination of organic additives. Simultaneously, there was negligible alteration observed in interparticle gaps, indicating an insignificant impact on the internal structure caused by trace amounts of organic additives.

#### 3.2.2. Density, Microstructure Evolution, and Phase Identification

In the previous work, a 45 vol% solids content was found to be suitable for 3D printing. To achieve transparency in the final product, three crucial factors were considered [33]: (1) enhancing density to eliminate porosity; (2) optimizing grain boundaries; and (3) ensuring homogeneous crystals without any second-phase impurities. In this section, these factors are evaluated through density evolution analysis, microstructure snapshot examination, and XRD characterization.

Compared to traditional CIP methods, generating pores in 3D-printed ceramic structures is facilitated. Pores are introduced through the removal of organics during calcination and the stacking of intermediate layers during printing. However, effective elimination of pores can be achieved by optimizing the sintering system. In this study, CIP and 3D-printed parts will be simultaneously sintered at three different temperatures (1750 °C, 1800 °C, 1850 °C) to investigate the influence of these temperatures on the density and properties of yttrium oxide transparent ceramics.

**Figure 7:**
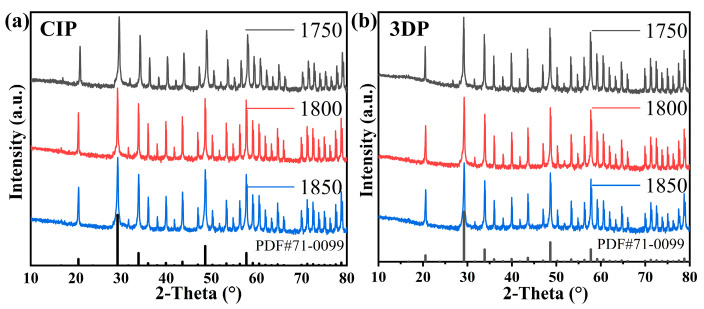
Phase identification. The XRD of (**a**) CIP and (**b**) 3D printing exhibit a single yttrium oxide phase. PDF cards from ICDD database: Yttrium Oxide #71-0099.

The sintered sample was further subjected to X-ray diffraction (XRD) analysis in order to investigate the crystalline phase of yttria. The obtained results are presented in Figure 7, which demonstrates a consistent presence of the yttria phase (PDF#71-0099) and indicates a high degree of crystallization based on the peak intensity. No second phases were observed in the XRD pattern.

**Figure 8:**
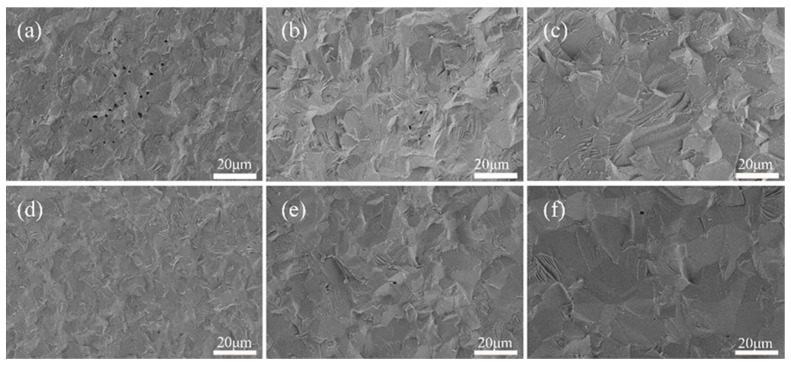
Scanning electron microscopy (SEM) images depicting the fracture surfaces of 3D printing samples sintered at varying temperatures: (**a**) 1750 °C, (**b**) 1800 °C, and (**c**) 1850 °C, as well as the fracture surface of cold isostatic pressed (CIP) samples sintered under varying temperature conditions: (**d**) 1750 °C, (**e**) 1800 °C, and (**f**) 1850 °C.

The process of densification is effectively demonstrated through the evolution of microstructure. The SEM images in Figure 8a–c depict the 3D-printed samples after sintering at temperatures of 1750 °C, 1800 °C, and 1850 °C, respectively, while SEM images in Figure 8d–f illustrate the CIP samples after sintering at the same temperatures. A comparison between the fracture surfaces of ceramic samples obtained from both 3D printing and CIP techniques was conducted to analyze their different microstructures. It should be noted that, due to the inherent characteristics of the DIW process and de-binding of additives, surface pores were observed on the sintered ceramic produced by 3D printing (Figure 8a). With the increase in the sintering temperature, the porosity of 3D printing gradually decreased, and at 1850 °C, the section exhibited negligible porosity. In CIP, there were virtually no pores observed at 1750 °C, accompanied by small and uniform grain sizes. As the sintering temperature rose further, rapid grain growth occurred, impeding timely pore elimination; consequently, some grain boundary pores transformed into intracrystalline pores due to abnormal grain growth. Consequently, an increase in sintering temperature led to an elevation in CIP porosity.

### 3.3. Optical Properties of Ceramic Objects

**Figure 9:**
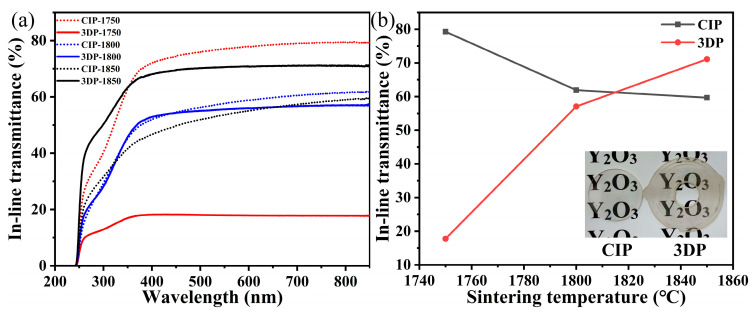
(**a**) In-line transmittance of 3D-printed and CIP ceramics post-sintering. (**b**) In-line transmittance of 3D-printed and CIP ceramics at varying sintering temperatures at 850 nm.

The optical properties of 3D printed ceramics were compared with CIP-shaped ceramics with a sample thickness of approximately 0.9 mm. Figure 9 shows the live effect of transmittance.

Within the visible region, the peak transmission of 3D printed ceramic was about 71%, lower than that of the CIP sample (79%). The gap between 3D-printed and CIP-shaped ceramics can be attributed to the pores originating from the decomposition of organic additives and the defects introduced by the DIW itself, such as layer-to-layer potential pores [18,25]. With increasing sintering temperature, the transmittance of 3D printing increased, while the transmittance of the CIP sample decreased. This is consistent with the inference from the SEM diagram that the porosity decreased with the increase in the sintering temperature of 3D printing ceramics, and more pores appeared in CIP samples [34].

## 4. Conclusions

The present study demonstrates the 3D printing of yttrium oxide transparent ceramics using the DIW technique. Detailed investigations on the rheological properties of the slurry, including additive content, pH, and solid loading, were conducted to design a high-solid-content paste (45 vol%) and a shear-thinning print slurry suitable for DIW. The resulting 3D-printed ceramic exhibited in-line transmittance of 71% at a sintering temperature of 1850 °C, which is slightly lower than that achieved by conventional CIP (79%). However, it enabled the fabrication of complex structures that are unattainable through CIP. It should be noted that post-processing steps such as grinding and polishing pose challenges for complex structures fabricated via 3D printing and require further attention and development. The DIW printing approach for yttrium oxide ceramics holds great potential to expand their applications in optics.

## Data Availability

Data are contained within the article.
